# A Kinase-Independent Role for Cyclin-Dependent Kinase 19 in p53 Response

**DOI:** 10.1128/MCB.00626-16

**Published:** 2017-06-15

**Authors:** K. Audrey Audetat, Matthew D. Galbraith, Aaron T. Odell, Thomas Lee, Ahwan Pandey, Joaquin M. Espinosa, Robin D. Dowell, Dylan J. Taatjes

**Affiliations:** a Department of Chemistry and Biochemistry, University of Colorado, Boulder, Colorado, USA; b Department of Molecular, Cellular, and Developmental Biology, University of Colorado, Boulder, Colorado, USA; c Department of Pharmacology and Linda Crnic Institute for Down Syndrome, School of Medicine, University of Colorado, Anschutz Medical Campus, Aurora, Colorado, USA; d BioFrontiers Institute, University of Colorado, Boulder, Colorado, USA

**Keywords:** CDK8, CDK19, Mediator kinase, p53, cholesterol, nutlin, nutlin-3, stress, transcription, drug resistance, 5-FU, 5-fluorouracil, SJSA, RNA-Seq, cortistatin A, osteosarcoma

## Abstract

The human Mediator complex regulates RNA polymerase II transcription genome-wide. A general factor that regulates Mediator function is the four-subunit kinase module, which contains either cyclin-dependent kinase 8 (CDK8) or CDK19. Whereas CDK8 is linked to specific signaling cascades and oncogenesis, the cellular roles of its paralog, CDK19, are poorly studied. We discovered that osteosarcoma cells (SJSA) are naturally depleted of CDK8 protein. Whereas stable CDK19 knockdown was tolerated in SJSA cells, proliferation was reduced. Notably, proliferation defects were rescued upon the reexpression of wild-type or kinase-dead CDK19. Comparative RNA sequencing analyses showed reduced expression of mitotic genes and activation of genes associated with cholesterol metabolism and the p53 pathway in CDK19 knockdown cells. SJSA cells treated with 5-fluorouracil, which induces metabolic and genotoxic stress and activates p53, further implicated CDK19 in p53 target gene expression. To better probe the p53 response, SJSA cells (shCDK19 versus shCTRL) were treated with the p53 activator nutlin-3. Remarkably, CDK19 was required for SJSA cells to return to a proliferative state after nutlin-3 treatment, and this effect was kinase independent. These results implicate CDK19 as a regulator of p53 stress responses and suggest a role for CDK19 in cellular resistance to nutlin-3.

## INTRODUCTION

Cyclin-dependent kinase 19 (CDK19) and its paralog CDK8 are kinases that reversibly associate with the Mediator complex (1). Whereas CDK8 is conserved in eukaryotes (Srb10 gene in Saccharomyces cerevisiae), CDK19 emerged in vertebrates and relatively little is known about CDK19 versus CDK8. The amino acid sequence of CDK19 is 77% identical (82% similar) to CDK8, with 97% identity in the kinase domain. As Mediator-associated kinases, CDK8 and CDK19 interact with Mediator as part of a four-subunit, 600-kDa complex called the kinase module (also known as CDK8 module with CDK8 or CDK19 module with CDK19). The other subunits within the kinase module are MED12 (or its paralog MED12L), MED13 (or its paralog MED13L), and CCNC (2). Chromatin immunoprecipitation sequencing data indicate that kinase module components colocalize with Mediator subunits genomewide (3–6), and global targeting of the kinase module appears to reflect its association with Mediator. Although kinase module-Mediator association is reversible (7–9), the association is stable, since a distinct pool of “CDK8-Mediator” complexes exists in cells and can be biochemically purified (10). In fact, in proliferating human cells, Mediator appears to exist in primarily two distinct forms: a core complex (here simply called Mediator) and a CDK-Mediator complex, which includes the kinase module (containing CDK8 or CDK19).

Mediator is a central regulator of RNA polymerase II (Pol II) transcription and is recruited to enhancer and promoter regions by DNA-binding transcription factors (TFs) (1). Mediator interacts extensively with the Pol II enzyme (11–14) and is important for communicating regulatory signals from DNA-binding TFs to the Pol II transcription machinery. The interaction of the kinase module with Mediator appears to act as a switch that regulates Mediator–Pol II association (15–17). In particular, Mediator interaction with the CDK8 module or Pol II appears to be mutually exclusive; CDK8-Mediator will not interact with Pol II, and Mediator-Pol II assemblies will not interact with the CDK8 module (16–19). Consequently, global gene expression patterns may rely upon precise regulation of the kinase module-Mediator association. Although this suggests a genome-wide requirement for CDK8 or CDK19, the kinase module-Mediator association is actually regulated by the MED13 subunit (16, 17), and knockdown of CDK8 or CDK19 in human cells causes relatively modest effects on gene expression (3, 20, 21).

In addition to directly regulating Mediator function, Mediator kinases may facilitate transcription regulation via cell signaling cascades. CDK8 has been linked to the KRAS, Notch, gamma interferon (IFN-γ), and Wnt/β-catenin signaling pathways in mammalian cells (22–25), and a growing number of studies link the Mediator kinase module to developmental diseases and specific types of cancer (26–34). Moreover, CDK8 appears to have ancient links to metabolism that appear to derive from transcriptional regulation of metabolic genes (35, 36) and phosphorylation of nutrient-responsive TFs (37, 38).

Because relatively little is known about CDK19 compared to CDK8, we sought to better understand CDK19 function in human cells. To facilitate this analysis, we identified a cancer cell line (SJSA) that is naturally depleted of CDK8 protein but retains CDK19. Our analysis revealed novel links between CDK19 and cell proliferation, p53 response, and cholesterol metabolism. CDK19 appeared to contribute to repression of p53 target genes under basal conditions, and CDK19 was important for maximal induction of stress response genes, including p53 targets, after treatment with the genotoxic agent 5-fluorouracil (5-FU). Moreover, SJSA proliferation was blocked in CDK19 knockdown cells after treatment with the p53 activator nutlin-3, whereas control cells recovered to a proliferative state. These results suggest CDK19 regulates the p53 transcriptional response and implicate CDK19 in cell proliferation and cholesterol metabolism. Notably, such roles have been linked to CDK8 in other cell types.

## RESULTS

### SJSA cells as a model to study CDK19.

**FIG 1:**
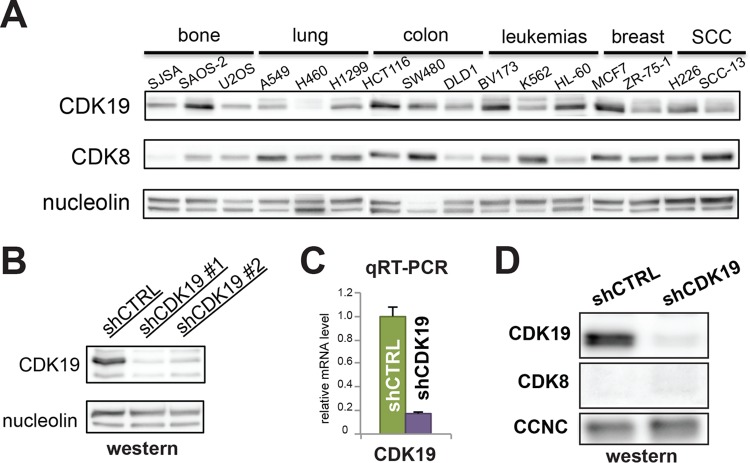
SJSA cells lack CDK8 protein but retain CDK19. (A) Western analysis of multiple cancer cell lines from a variety of tissue types. CDK19 levels vary among cell types, whereas CDK8 is consistently expressed, except in SJSA cells. (B and C) Western (B) and qRT-PCR (C) analyses showing the levels of CDK19 protein and mRNA in control knockdown (shCTRL) or CDK19 knockdown (shCDK19) SJSA cells. (D) Western data confirming that CDK8 protein levels are not induced upon CDK19 knockdown.

Western blot analyses of multiple cancer cell types revealed that most express both CDK8 and CDK19 protein. SJSA cells, an osteosarcoma cell line, contain CDK19, but the CDK8 protein is almost undetectable (Fig. 1A). Given this characteristic, SJSA cells represented a good model system for evaluating the functional role of CDK19 by minimizing potential interference by its paralog, CDK8.

### Reduced proliferation in SJSA cells upon stable CDK19 knockdown.

**FIG 2:**
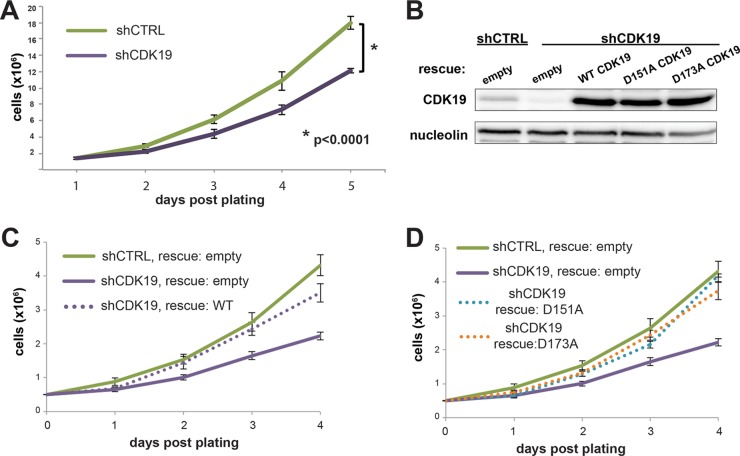
SJSA cell growth is inhibited upon CDK19 knockdown but rescued with WT or kinase-dead CDK19. (A) Growth curve of shCTRL and shCDK19 SJSA cells under normal growth conditions. (B) Western blot showing expression levels of CDK19 proteins following transfection with indicated plasmids. (C and D) Transient expression of wild-type CDK19 (C) or kinase-dead CDK19 (D; D151A or D173A) rescues the lowered growth rate of shCDK19 cells.

Given the lack of CDK8 protein, we suspected that knockdown of CDK19 might cause widespread cell death in SJSA cells. However, we were able to establish a stable CDK19 knockdown cell line (Fig. 1B and C), along with a control line expressing a nontargeting shRNA, suggesting that CDK19 is not essential for SJSA cell survival. Upon CDK19 knockdown, CDK8 levels remained unchanged and essentially undetectable in SJSA cells (Fig. 1D). CDK19 knockdown cells (shCDK19) remained viable but exhibited a lower growth rate than the control cells (shCTRL) (Fig. 2A). To confirm that the growth defect was due to loss of CDK19 and not an off-target effect, we tested whether growth could be “rescued” by expression of wild-type (WT) CDK19 from cDNA lacking the 3′-untranslated-region sequence targeted by the CDK19 shRNA (Fig. 2B). As shown in Fig. 2C, the growth rate of CDK19 knockdown cells expressing wild-type CDK19 (shCDK19 rescue: wild type) matched the growth rate of shCTRL cells “rescued” with an empty vector (shCTRL rescue: empty). These results indicated that reduced proliferation was due to loss of the CDK19 protein and not an off-target effect of shRNA expression.

We next assessed whether the growth defect in shCDK19 SJSA cells was due primarily to loss of CDK19 kinase activity, or loss of the CDK19 protein *per se*. We generated two shRNA-resistant CDK19 kinase-dead mutants, D151A and D173A (Fig. 2B), which mutate the conserved proton acceptor and activation loop residues in the kinase domain, respectively (39). Both mutants rescued the growth rate to a similar extent as the wild-type protein (Fig. 2D), suggesting that the physical presence of the CDK19 protein, not its kinase activity, was required to maintain the proliferation rate under basal growth conditions.

### CDK19 knockdown affects mRNA levels of p53 pathway, cholesterol homeostasis, and mitotic genes.

**FIG 3:**
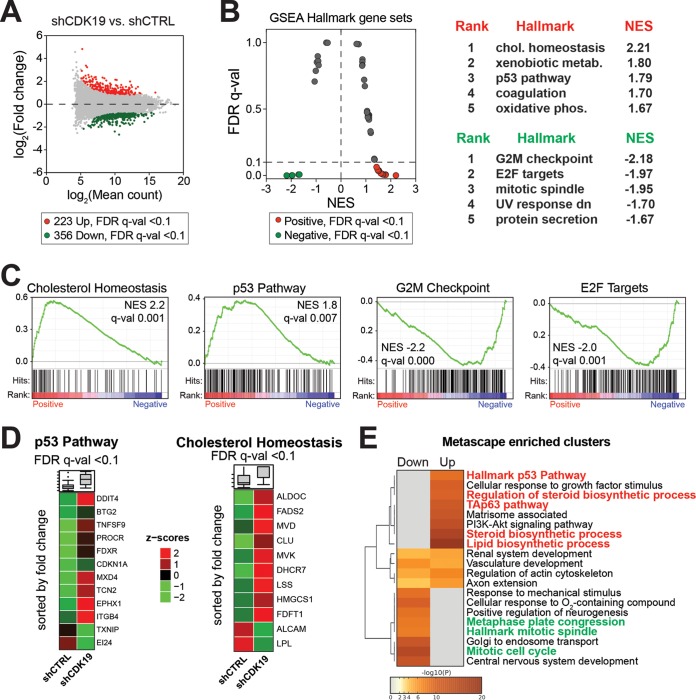
Gene expression (RNA-Seq) changes due to CDK19 knockdown affect the p53 pathway, mitosis, and cholesterol homeostasis. (A) MA plot comparing control and CDK19 knockdown SJSA cells. (B) Plot of false discovery rate (FDR) versus the normalized enrichment score (NES) based upon GSEA from RNA-Seq data (shCDK19 versus shCTRL). The dashed line represents the 0.1 FDR cutoff. Note that the p53 pathway and cholesterol homeostasis are positively enriched in CDK19 knockdown cells, whereas gene sets associated with mitosis are negatively enriched. The top five ranked positively and negatively enriched gene sets are shown on the right. (C) GSEA plots for selected hallmark gene sets, with black bars indicating gene sets represented among all genes ranked by log_2_-fold change (shCDK19 versus shCTRL). (D) Heat maps showing average expression, calculated from the reads per kilobase per million (RPKM), of the p53 pathway and cholesterol homeostasis genes; only genes meeting an FDR *q* < 0.1 threshold are shown. (E) Heat map of Metascape-enriched clusters. Each cluster contains multiple gene sets to eliminate redundancy. Analysis used genes meeting an FDR *q* < 0.1 threshold.

We examined global gene expression changes due to CDK19 knockdown with comparative RNA sequencing (RNA-Seq) analyses, each in duplicate (see Table S2 in the supplemental material). The expression of approximately 675 genes was significantly changed (false-discovery rate [FDR] *q* < 0.1) in CDK19 knockdown cells compared to nontargeting shRNA controls (Fig. 3A and Table S3 in the supplemental material). Based upon the RNA-Seq data, we ranked all genes by their fold change between CDK19 knockdown versus control cells and performed gene set enrichment analysis (GSEA) (40) to identify hallmark gene sets enriched among the most up- or downregulated mRNAs. Genes associated with cholesterol homeostasis and the p53 pathway were enriched among those with increased expression in CDK19 knockdown cells compared to control cells, whereas gene sets associated with mitosis (G_2_M checkpoint, E2F targets) tended to show decreased expression (Fig. 3B and C; see also Table S4 in the supplemental material). Heat maps of genes with significant changes (FDR *q* < 0.1) in the cholesterol homeostasis or p53 pathway gene sets that were affected by CDK19 knockdown are shown in Fig. 3D. We also completed analysis with Metascape (41), which tests for overrepresentation of a larger number of pathways and gene sets (including those covered by GSEA) and reduces redundancy by clustering enriched terms based upon the degree of shared genes (see Table S5 in the supplemental material). A heat map of Metascape-enriched clusters for down- or upregulated genes (FDR *q* < 0.1; shCDK19 versus shCTRL) is shown in Fig. 3E. As with the GSEA analysis (Fig. 3B), Metascape-enriched gene clusters showed significant upregulation of p53 pathway genes and downregulation of mitosis genes upon CDK19 knockdown. Reduced proliferation observed in CDK19 knockdown cells (Fig. 2A) could result from reduced expression of mitosis genes; however, basal activation of p53 pathway genes could also contribute, since p53 activates the expression of several cell cycle inhibitors.

### The transcriptional response to 5-FU is dampened upon CDK19 knockdown.

Despite the lack of detectable CDK8 protein, the effect of stable CDK19 knockdown only modestly affected SJSA cell proliferation. We postulated that the effects of CDK19 knockdown might manifest more severely under cell stress. To test this idea, we treated control versus shCDK19 cells with the cytotoxic anti-metabolite drug 5-fluorouracil (5-FU). This was based in part upon previous results in 5-FU-treated HCT116 cells that showed greater gene expression changes with CDK19 knockdown versus CDK8 knockdown (3). Notably, the phenotypic differences between control and CDK19 knockdown cells were again quite modest. Although CDK19 knockdown cells proliferated more slowly than the shCTRL cells, the rate was not impacted by 5-FU treatment (12 h); both shCDK19 and shCTRL cells began to recover a few days after 5-FU treatment (data not shown).

**FIG 4:**
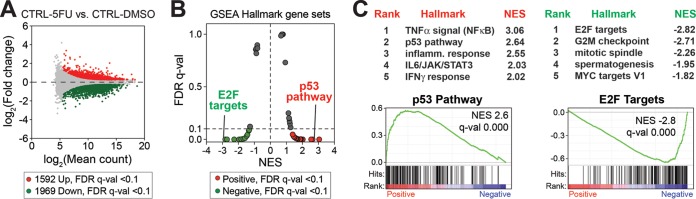
SJSA transcriptional response to 5-FU. (A) MA plot comparing RNA-Seq data from shCTRL SJSA cells after 5-FU treatment (versus DMSO control). (B) Plot of FDR versus the normalized enrichment score (NES) based upon GSEA from RNA-Seq data (shCTRL cells, 5-FU versus DMSO). The dashed line represents 0.1 FDR cutoff. As expected, stress response (e.g., p53 pathway) gene sets are enriched, whereas proliferative gene sets (e.g., G_2_/M checkpoint) are reduced in 5-FU treated cells. (C) Top five ranked positively and negatively enriched gene sets and GSEA plots for p53 pathway and E2F targets.

To assess potential gene expression changes in shCTRL and shCDK19 cells in response to 5-FU, we completed RNA-Seq experiments following a 12-h 5-FU treatment. The RNA-Seq experiments completed in shCTRL or shCDK19 cells treated with dimethyl sulfoxide (DMSO; vehicle for 5-FU) (Fig. 3) served as basal controls. In the shCTRL cells, a strong transcriptional response to 5-FU was observed (see Table S2 in the supplemental material); 1,592 genes were upregulated, and 1,969 genes were downregulated (5-FU versus DMSO; FDR *q* < 0.1) (Fig. 4A and Table S3 in the supplemental material). As expected, GSEA (Fig. 4B and C and Table S4 in the supplemental material) revealed gene signatures related to stress responses (e.g., inflammation, IFN-γ, and the p53 pathway) were markedly enriched in 5-FU-treated cells, whereas gene sets associated with cell proliferation (e.g., G_2_/M checkpoint, mitotic spindle, etc.) were negatively affected.

**FIG 5:**
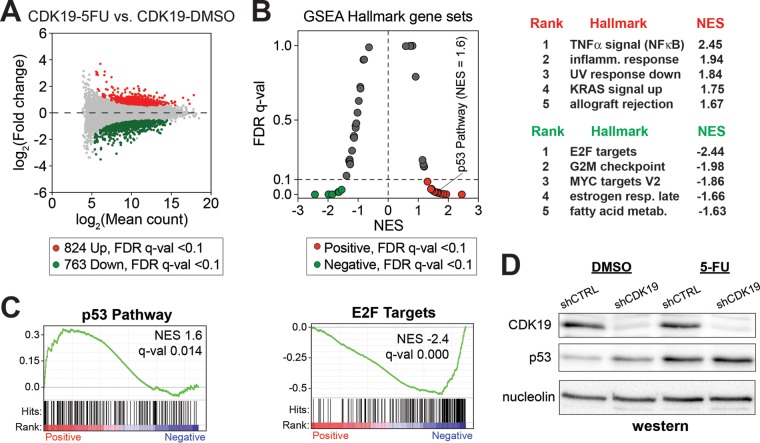
Transcriptional response to 5-FU is dampened in CDK19 knockdown cells. (A) MA plot comparing RNA-Seq data from shCDK19 SJSA cells after 5-FU treatment (versus DMSO control). Compared to shCTRL cells (Fig. 4A), the shCDK19 cells show an overall decreased transcriptional response. (B) Plot of FDR versus the NES based upon GSEA from RNA-Seq data (shCDK19 cells, 5-FU versus DMSO). The dashed line represents 0.1 FDR cutoff. The top five ranked positively and negatively enriched gene sets are shown at right. (C) GSEA plots for p53 pathway and E2F targets. (D) Western blot showing expression levels of CDK19 and p53 in the control and CDK19 knockdown SJSA cells after 5-FU treatment.

In contrast, global gene expression changes were muted in CDK19 knockdown cells (5-FU versus DMSO) (Fig. 5A and Table S2 in the supplemental material). Given the same 5-FU treatment, 824 genes were upregulated, and 763 genes were downregulated in CDK19 knockdown cells (see Table S3 in the supplemental material). Collectively, this represented an ∼2-fold reduction in the number of genes induced or repressed upon 5-FU treatment. Furthermore, GSEA showed reduced enrichment of gene sets in 5-FU-treated shCDK19 cells compared to shCTRL cells (versus DMSO controls) (Fig. 5B and C and Table S4 in the supplemental material). For instance, the normalized enrichment scores (NES) for p53 pathway and inflammatory response were reduced in 5-FU treated shCDK19 cells (compare the NES in Fig. 4C and 5B). As expected, 5-FU increased the p53 protein levels in both shCTRL and shCDK19 SJSA cells (Fig. 5D).

**FIG 6:**
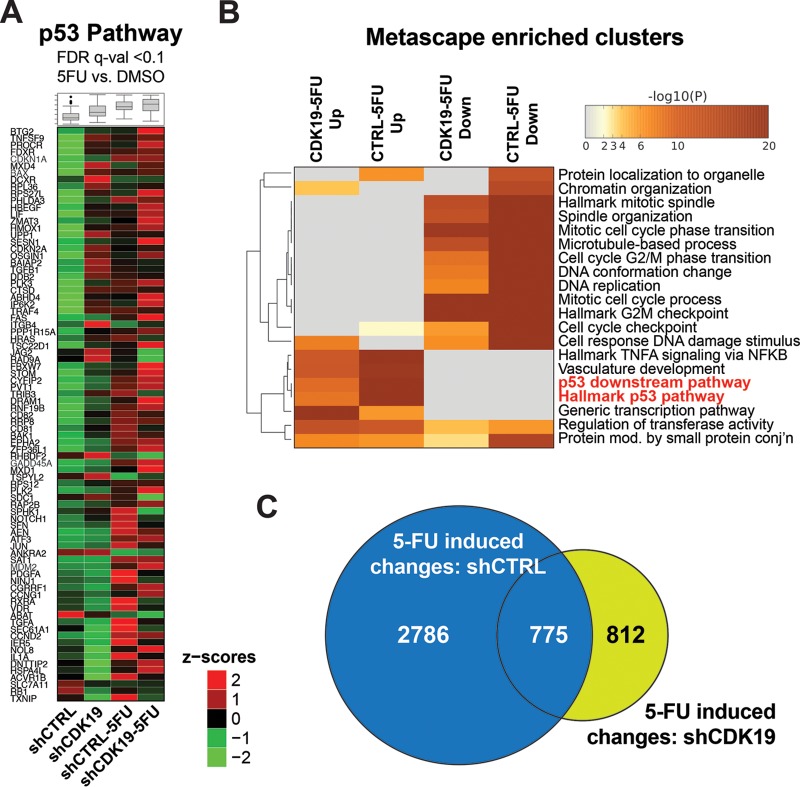
Differential p53 pathway activation in shCDK19 cells. (A) Heat maps showing average expression (calculated from the RPKM) of p53 pathway genes under conditions tested in this study; only genes meeting an FDR *q* < 0.1 threshold are shown. (B) Heat map of Metascape-enriched clusters (each cluster contains multiple gene sets to eliminate redundancy) in 5-FU-treated cells (shCDK19 or shCTRL), separated by up- or downregulated. Analysis used genes meeting an FDR *q* < 0.1 threshold. (C) Venn diagram showing overlap of differentially expressed genes in shCTRL versus shCDK19 cells upon 5-FU treatment (i.e., shCTRL data from Fig. 4A and shCDK19 data from Fig. 5A).

Expanded analysis of p53 pathway gene induction in shCTRL and shCDK19 cells is shown in Fig. 6A and B (see also Table S5 in the supplemental material), which further supports altered p53 transcriptional responses in shCDK19 cells. However, it was notable that p53 target genes showed varied effects in shCDK19 cells (versus shCTRL) under basal or stressed (5-FU) conditions. That is, a subset of p53 pathway genes showed increased mRNA levels in shCDK19 cells (versus shCTRL) under basal conditions or after treatment with 5-FU. Moreover, although the RNA-Seq data suggested a reduced overall transcriptional response to 5-FU in shCDK19 cells (compare Fig. 5A to C with Fig. 4A to C), only a subset of the genes induced during 5-FU treatment were identical (Fig. 6C). Thus, distinct sets of genes were induced in shCDK19 cells in response to 5-FU. Taken together, the data in Fig. 4 to 6 suggested that CDK19 plays an important role in regulating the transcriptional response to 5-FU in SJSA cells.

### CDK19 knockdown sensitizes SJSA cells to the p53 activator nutlin-3.

RNA-Seq experiments and pathway analysis with GSEA or Metascape revealed that p53 target gene expression increased during basal conditions in shCDK19 versus shCTRL cells (Fig. 3B to E). Moreover, induction of p53 target genes was altered in 5-FU-treated shCDK19 cells, relative to controls (e.g., compare NES in Fig. 4C to Fig. 5B). These results suggested that the p53 pathway might be unusually sensitive to CDK19 protein levels in SJSA cells. We decided to pursue this further by treating SJSA cells with nutlin-3, an exquisitely selective activator of p53 (42).

**FIG 7:**
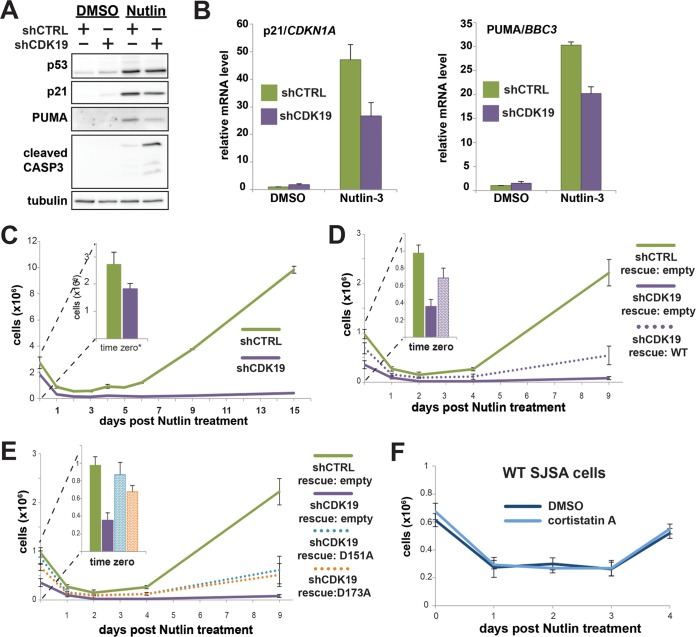
CDK19 knockdown sensitizes SJSA cells to nutlin-3. (A) Western blot data showing stabilization of p53 protein in nutlin-3-treated cells; p21, PUMA, and cleaved caspase-3 also induced in nutlin-treated shCTRL or shCDK19 cells. (B) qRT-PCR analyses confirm that nutlin-3 induces p53 target gene expression and reveal that, as suggested in 5-FU-treated cells, induction of select p53 targets is diminished in shCDK19 cells. (C) Cell proliferation following 24 h of nutlin-3 treatment (shCTRL or shCDK19). Whereas shCTRL cells recover to a proliferative state, shCDK19 cells do not (inset: cell counts immediately after nutlin-3 treatment). (D and E) Rescue expression of WT CDK19 (D) or kinase-dead versions of CDK19 (E) reestablish cell proliferation after nutlin-3 treatment, indicating that the physical presence of CDK19 is important for SJSA cells to return to a proliferative state following nutlin-3 treatment. (F) The Mediator kinase inhibitor CA, which inhibits both CDK8 and CDK19 (5), does not negatively affect SJSA cell recovery following nutlin-3 treatment, further implicating the CDK19 protein, not its kinase activity *per se*, as the underlying cause for the nutlin sensitivity.

Nutlin-3 activates p53 by inhibiting its interaction with HDM2, an E3 ubiquitin ligase that regulates degradation of p53. SJSA cells contain wild-type p53 but also possess abnormally high levels of HDM2, which effectively inactivates p53 and contributes to their cancerous state (43). We verified that nutlin-3 increased steady-state p53 protein levels in both shCTRL and shCDK19 knockdown cells, and we also noted increased levels of cleaved caspase-3 (a marker for apoptosis) in shCTRL and shCDK19 cells (Fig. 7A). These results are in agreement with previous experiments that showed induction of apoptosis in SJSA cells upon treatment with nutlin-3 (42). We next evaluated p53 target gene induction in shCTRL versus shCDK19 cells. As shown in Fig. 7B, reduced induction of p21/CDKN1A and PUMA/BBC3 was observed in nutlin-treated shCDK19 cells versus controls. Although only two p53 target genes were examined here, the results were in general agreement with data from 5-FU-treated SJSA cells that showed somewhat reduced activation of p53 target genes with CDK19 knockdown (e.g., compare NES in Fig. 4C to Fig. 5B). We emphasize, however, that in both 5-FU and nutlin-treated shCDK19 cells, p53 target genes could still be induced, but the level of induction did not match shCTRL cells.

We next tracked shCTRL versus shCDK19 cell populations during and after nutlin-3 treatment. As shown in Fig. 7C, nutlin-3 treatment resulted in a large decrease in the number of viable cells; however, over time the shCTRL cells recovered and proliferated, whereas the shCDK19 cells did not. Additional experiments (data not shown) that used shorter nutlin-3 treatment times (12 h) or multiple treatments showed similar trends: shCDK19 cells were more sensitive to nutlin-3 compared to shCTRL SJSA cells.

To confirm that the difference in nutlin-3 sensitivity was due to reduced CDK19 protein levels and not potential off-target effects of the CDK19 shRNA, we again completed studies in shCDK19 SJSA cells with “rescue” expression of an shRNA-resistant CDK19 (Fig. 2B). SJSA shCDK19 cells with exogenous CDK19 expression were able to recover from nutlin-3 treatment (Fig. 7D). Although the recovery of the CDK19 “rescue” population did not match that of the shCTRL cells, this likely reflected the incomplete transfection efficiency of the CDK19 rescue experiments (the efficiency was determined to be ∼24%). Taken together with the RNA-Seq data, these results indicated that the CDK19 protein is an important regulator of the p53 pathway.

### The physical presence of CDK19 protein, not its kinase activity, restores SJSA proliferation after nutlin-3 treatment.

We next sought to determine whether CDK19 kinase activity was essential for the phenotypic change observed in shCDK19 SJSA cells after nutlin-3 treatment. CDK19 knockdown cells were transfected with shRNA-resistant vectors that expressed kinase-dead mutant versions of CDK19 (D151A or D173A) (Fig. 2B). The shCDK19 cells with rescue expression of kinase-dead versions of CDK19 were able to recover from nutlin-3 treatment (Fig. 7E). In fact, the proliferation of the kinase-dead and wild-type CDK19 rescue cell populations were similar (compare Fig. 7D and E). As with the wild-type CDK19 rescue experiments (Fig. 7D), the reduced proliferation compared to shCTRL cells likely derived from incomplete transfection efficiency of the shRNA-resistant rescue CDK19 expression vector (the efficiency was determined to be ∼24%).

As an additional control for CDK19 kinase activity, we treated wild-type SJSA cells with cortistatin A (CA) (5), a potent and highly selective inhibitor of CDK19. As shown in Fig. 7F, SJSA cell recovery after treatment with nutlin-3 was identical in CA versus DMSO control cells. Because CA inhibits CDK8 and CDK19 equally well (5), these data also indicate that potential confounding effects from low levels of CDK8 kinase activity are not contributing to proliferation defects following nutlin-3 treatment in shCDK19 cells (but see below). We conclude from the data in Fig. 7 that the CDK19 protein, but not its kinase activity, is required for SJSA cells to return to a proliferative state after nutlin-3 treatment.

### CDK19 is recruited to p53 target genes, but knockdown does not markedly affect p53 or Pol II occupancy.

**FIG 8:**
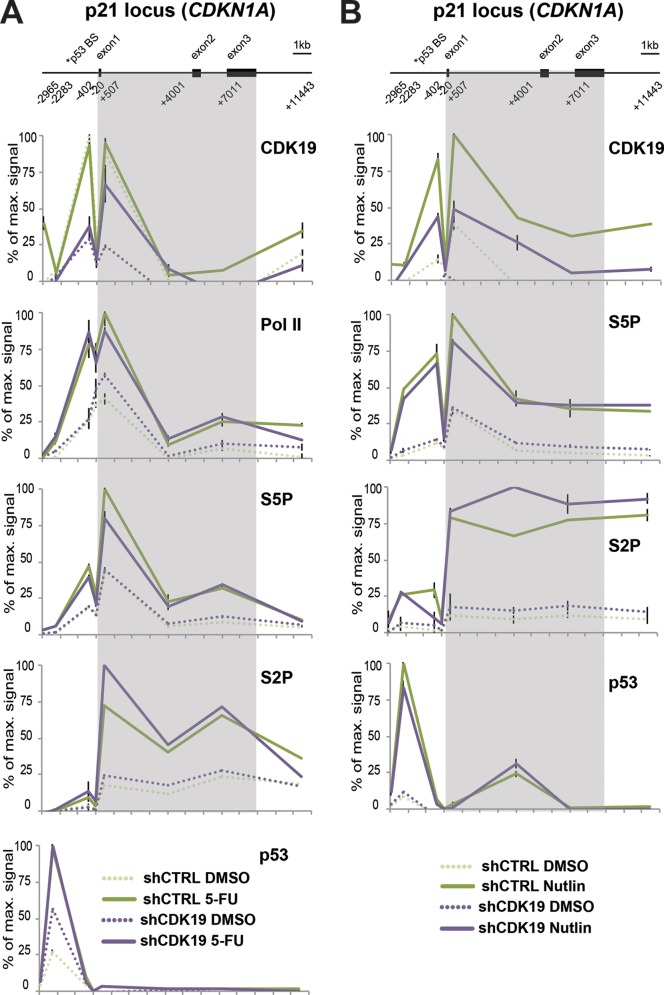
CDK19 associates with p53 target gene CDKN1A; knockdown does not notably affect Pol II or p53 occupancy. (A) ChIP data comparing DMSO- and 5-FU-treated cells. A scheme of the p21 locus is shown at the top. Numbers indicate primer positions used for gene tiling. Positions are given as the distance from the transcription start site. Proteins probed by ChIP are shown at the upper right of each plot. The key for each experiment is shown at the bottom. (B) ChIP data comparing DMSO- and nutlin-treated cells (6 h treatment). The scheme of the p21 locus is shown at the top. Numbers indicate primer positions used for gene tiling. Positions are given as the distance from the transcription start site. Proteins probed by ChIP are shown at the upper right of each plot. The key for each experiment is shown at bottom.

Because knockdown cell lines take time to develop, the effects observed in CDK19 knockdown cells could result from CDK19 directly and/or indirectly influencing transcription. To begin to assess whether CDK19 might directly affect expression of p53 target genes, we completed ChIP assays at the p21/CDKN1A locus under normal (DMSO) conditions or following 5-FU (Fig. 8A) or nutlin-3 (Fig. 8B) treatment. The data suggest that CDK19 occupies the p21 locus in SJSA cells (note the reduced CDK19 occupancy upon knockdown), but knockdown does not notably affect occupancy of p53 or Pol II. However, because the changes in steady-state levels of p53 target genes are modest under these circumstances (i.e., 5-FU and nutlin-3 still induce p21/CDKN1A expression in shCDK19 cells, just to a lower level versus shCTRL cells), it may be difficult to observe clear differences in Pol II occupancy. These data may instead reflect potential cotranscriptional or posttranscriptional regulation by CDK19. The precise mechanism(s) by which CDK19 may affect mRNA levels of p53 target genes remains to be determined.

### Cholesterol levels do not appear to affect SJSA sensitivity to nutlin-3.

**FIG 9:**
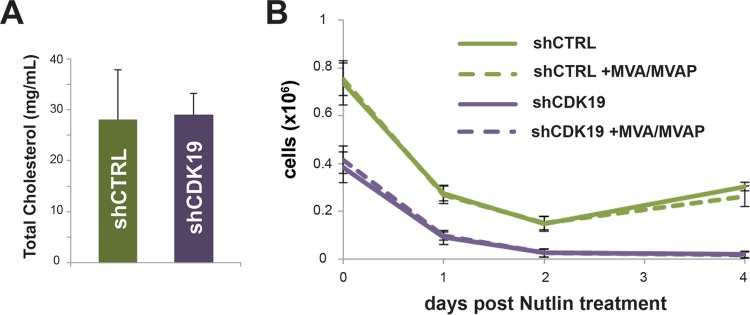
Analysis of cholesterol in shCTRL versus shCDK19 cells. (A) Total cholesterol levels in each cell line. (B) Supplementation of culture media with MVA and MVAP, which are cholesterol biosynthesis intermediates, did not affect cell recovery after treatment with nutlin-3.

In addition to p53, the RNA-Seq data indicated that cholesterol and lipid homeostasis genes were upregulated in CDK19 knockdown versus control (shCTRL) SJSA cells (Fig. 3B to D). To probe this further, we measured global cholesterol levels in control versus shCDK19 cells but observed no difference (Fig. 9A). Moreover, we assessed whether manipulation of cellular cholesterol levels would affect SJSA cell recovery after treatment with nutlin-3. To increase cholesterol biosynthesis intermediates, we treated SJSA cells (shCTRL or shCDK19) with mevalonic acid (MVA) and mevalonic acid phosphate (MVAP), essentially as described previously (44). As shown in Fig. 9B, treatment with MVA plus MVAP did not cause any notable affect on SJSA cell recovery after nutlin-3 treatment. As seen previously, the shCDK19 cells were unable to recover to a proliferative state (blue line, Fig. 9B), whereas the shCTRL cells began to proliferate about 48 h after treatment (green line, Fig. 9B). We also attempted to decrease cholesterol biosynthesis by treating cells with simvastatin as described previously (44). However, simvastatin treatment alone caused cell death and prevented an accurate assessment of its effects in nutlin-treated cells. Thus, although expression of genes involved in cholesterol homeostasis was clearly affected by CDK19 knockdown (Fig. 3B to D), cholesterol levels do not appear to be different in shCDK19 versus shCTRL SJSA cells. Furthermore, manipulation of cholesterol biosynthesis via MVA/MVAP did not notably affect SJSA cell recovery after nutlin-3 treatment.

### CDK8 rescues proliferation defect in shCDK19 cells and partially restores SJSA proliferation after nutlin-3 treatment.

**FIG 10:**
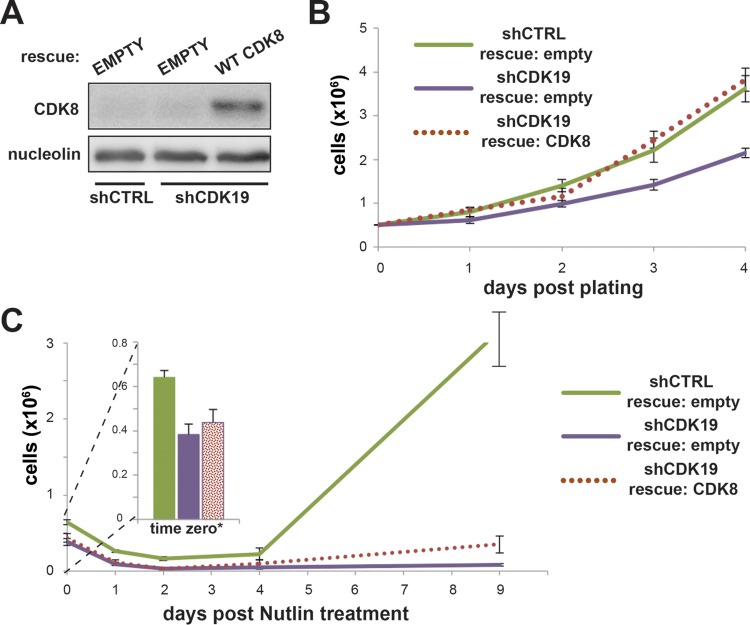
CDK8 can rescue growth defects in shCDK19 cells. (A) Western blot showing expression levels of CDK8 after transfection with empty vector or CDK8 expression plasmids. (B) Expression of CDK8 rescues the lowered growth rate of shCDK19 cells. (C) Expression of CDK8 rescues the proliferation defect in shCDK19 cells treated with nutlin-3. A low transfection efficiency (estimated to be ∼24%) likely contributes to the inability of the rescue to match shCTRL cells.

CDK8 is a highly similar paralog of CDK19, with 97% sequence identity in the kinase domain. Although the focus of this study was CDK19, we tested whether CDK8 might similarly “rescue” proliferation defects in shCDK19 cells. Whereas few studies have tested CDK8 versus CDK19 directly, there is evidence both for (5) and against (3) redundant functions for these Mediator-associated kinases. Interestingly, we observed that expression of CDK8 (Fig. 10A) in shCDK19 SJSA cells was able to rescue the general proliferation defect (Fig. 10B) and was able to restore SJSA proliferation following nutlin-3 treatment (Fig. 10C). The extent of proliferation rescue, however, appeared reduced compared to the rescue of CDK19 expression (compare Fig. 10C to Fig. 7D). (Due to the transfection efficiency of ∼24%, the CDK8 rescue experiments were not expected to match recovery in shCTRL cells.) Collectively, these data suggest that CDK8 and CDK19 have overlapping functions in SJSA cells, at least under the conditions studied here.

## DISCUSSION

In an effort to better delineate CDK19-specific roles from potential redundant functions of CDK8, we screened cancer cell lines to assess whether any might serve as a useful “model system” to study CDK19. We observed through Western blotting that SJSA cells have CDK8 protein that is barely detectable, whereas CDK19 is abundant. SJSA cells are derived from a 19-year-old patient with osteosarcoma. Although osteosarcomas are genetically diverse with distinct oncogenic drivers, a majority possess mutations that inactivate the p53 pathway and/or the RB1 transcription factor (45, 46). A subset of osteosarcomas contain deletions in the 6q16-6q23 chromosomal region (47), which contains both CCNC and CDK19. The link between p53 activation (tumor suppression) and the absence of CDK19 described here suggested that osteosarcomas bearing deletions in chromosome 6q16-6q23 may selectively possess p53 gain-of-function mutations; however, no correlation could be found among the few documented chromosomal deletion samples.

The biological functions for CDK19 are likely to be cell-type- and context-specific, based upon current understanding of its paralog CDK8 (3, 20). Although CDK19 is less well studied than CDK8, current evidence supports both overlapping and nonredundant roles in transcription regulation and cell physiology. An example supporting redundant functions for CDK8 and CDK19 was reported in acute myeloid leukemia cells treated with the CDK8/CDK19 kinase inhibitor CA. CA treatment blocked proliferation and induced apoptosis, but growth could be restored upon expression of CA-resistant CDK8 or CDK19 alleles (5). Conversely, comparison of gene expression changes in CDK8- or CDK19-knockdown HCT116 cells revealed distinct sets of affected genes with overlaps that changed with cell context (e.g., normal growth versus hypoxia) (3).

RNA-Seq data in shCDK19 SJSA cells (versus shCTRL) suggested that CDK19 selectively regulates cholesterol homeostasis genes. Past studies have linked CDK8, a paralog of CDK19, to cholesterol metabolism. Expression of cholesterol biosynthesis genes was reduced upon CDK8 knockdown in Drosophila and mouse models, with potential links to the transcription factor SREBP (48). Similarly, inhibition of CDK8/CDK19 kinase activity with CA disproportionately affected protein levels of cholesterol metabolism genes in HCT116 cells (49). These results suggest that the Mediator kinases (CDK8 and CDK19) may regulate cholesterol metabolism in distinct cell types and perhaps across model organisms.

In the case of enzymes such as the CDK19 kinase, it is often observed that phenotypic changes due to protein knockdown/knockout are distinct from targeted enzyme inhibition. For example, experiments in mouse models have shown that knockout of the Gcn5 acetyltransferase or the Hdac2 deacetylase do not phenocopy catalytically inactive mutants of these enzymes (50, 51). SJSA cells showed reduced proliferation upon CDK19 knockdown. To assess whether proliferation defects were due to reduced CDK19 kinase activity or simply from the physical loss of CDK19, we completed “rescue” experiments in which shRNA-resistant wild-type or kinase-dead CDK19 mutants were transiently expressed. The results indicated that the physical presence of CDK19 drives the phenotypic change, both in normal growth and nutlin-induced conditions. These findings point to key structural/scaffolding roles for the CDK19 protein and are in general agreement with RNA-Seq data from HCT116 cells that compared the effects of Mediator kinase inhibition with CDK8 or CDK19 knockdown (49). Further studies will be required to assess the mechanisms whereby CDK19 regulates expression of stress response genes after treatment with 5-FU or nutlin-3.

RNA-Seq data from 5-FU-treated SJSA cells implicated CDK19 in the overall transcriptional stress response. Widespread changes in the transcriptional response (for both up- and downregulated genes) were observed in shCDK19 cells compared to controls. Among the gene sets disproportionately affected by CDK19 knockdown after 5-FU treatment were p53 targets, suggesting a role for CDK19 in the p53 pathway. Interestingly, analogous findings were made in HCT116 cells upon CDK8 knockdown (HCT116 cells contain roughly equivalent levels of CDK8 and CDK19); in particular, p53 target gene induction was reduced in CDK8 knockdown cells (52). 5-FU is a broadly toxic compound that will activate multiple cellular stress response pathways; by contrast, nutlin-3 is exquisitely selective for p53 activation through inhibition of the p53 repressor HDM2 (42). Whereas SJSA cells possess wild-type p53, the HDM2 gene is amplified ∼25-fold, making these cells unusually sensitive to nutlin-3 treatment (42, 43). An expectation was that reduced p53 activation in nutlin-treated shCDK19 cells (versus controls) would minimize p53-induced cell death. Contrary to these expectations, nutlin-treated control or CDK19 knockdown SJSA cells responded similarly, suggesting that the altered p53 response remained sufficient to trigger apoptosis in shCDK19 cells. Remarkably, however, shCDK19 cells failed to recover to a proliferative state following 24-h nutlin-3 treatment, whereas control cells renewed proliferation within a few days.

These results could point to a role for p53 in setting up cells to recover to a proliferative state after nutlin-3 treatment. That is, the expression pattern of p53 target genes in nutlin-treated shCTRL cells (versus their altered expression in shCDK19 cells) may have roles beyond the stress response, including reestablishment of proliferation following stress. Because p53 can induce cell cycle arrest, the basal level of p53 activation observed in shCDK19 cells (versus shCTRL) may also contribute to reduced shCDK19 cell proliferation after nutlin-3 treatment. Alternately, the proliferation defect in nutlin-treated shCDK19 cells may derive from myriad other transcriptional and/or metabolic changes that result from CDK19 depletion; additional studies will be needed to address these questions.

Treatment options for osteosarcoma typically involve surgery coupled with chemotherapy; unfortunately, clinical outcomes for patients with osteosarcoma have not improved dramatically in the past few decades, and resistance to chemotherapy is common (53). The observation that SJSA cells with reduced CDK19 protein levels were unable to recover from nutlin-3 treatment has potential therapeutic importance. Whereas SJSA cells are sensitive to nutlin-3 (42), a subset are refractory to nutlin-3 and were able to recover and propagate. However, this resistance mechanism was blocked in shCDK19 cells, which invokes a “synthetic lethal” effect of CDK19 with nutlin-3 (54) and suggests that a manifestation of CDK19 knockdown—perhaps misregulation of the p53 pathway—blocks SJSA resistance to nutlin-3. Future experiments will test this hypothesis in SJSA and other nutlin-sensitive cell lines.

## MATERIALS AND METHODS

### Cells and tissue culture.

SJSA-1 cells were grown in 15-cm dishes in RPMI medium supplemented with 10% fetal bovine serum, and penicillin-streptomycin (Pen-Strep) or antibiotic-antimycotic solution (Gibco) and grown in 5% CO_2_ at 37°C. DMSO vehicle control (Sigma-Aldrich), nutlin-3a (Sigma-Aldrich), and 5-FU (Sigma-Aldrich) were used at 0.1%, 10.0 μM, and 375.0 μM, respectively, unless otherwise stated.

### shRNA mediated knockdown.

Commercially available shRNAs precloned into the pLKO.1-Puro vector (shCTRL and shCDK19) were obtained from the Functional Genomics Facility at the University of Colorado Anschutz Medical Campus. Oligonucleotide sequences for shRNAs are listed in Table S1A in the supplemental material. Lentiviral particles were produced in HEK293FT packaging cells. SJSA cells were transduced with 0.45-μm-pore-size-filtered viral supernatants and selected with puromycin (Sigma-Aldrich) at 1.0 μg/ml for 5 to 7 days.

### qRT-PCR.

After treatment, cells were harvested by scraping and rinsed in cold phosphate-buffered saline (PBS). Total RNA was harvested with TRIzol reagent (Ambion, catalog no. 15596-026) according to the manufacturer's instructions. First-strand cDNA was prepared using 1 μg of total RNA in a qScript cDNA synthesis kit (Quanta Biosciences catalog no. 95047-100), or a RevertAid first-strand cDNA synthesis kit (Thermo Scientific catalog no. K1622). cDNA was analyzed by a quantitative reverse transcription-PCR (qRT-PCR) absolute quantification method (SYBR Select; ABI) on a 7900HT (ABI) or a CFX384 real-time system (Bio-Rad) instrument. The primer sequences are listed in Table S1B in the supplemental material.

### RNA sequencing.

Total RNA was harvested directly from cell culture plates using 10 ml of TRIzol reagent per 15-cm plate. The medium was removed, and the cells were rinsed once with cold PBS. The cells were pipetted thoroughly in TRIzol to ensure a homogenous mixture, and RNA was prepped from 1 ml of TRIzol solution. After extraction with chloroform, the samples were precipitated with isopropanol, washed with ethanol, and cleaned using an RNeasy minikit (Qiagen). Samples were resuspended in water and taken directly to the Genomics and Microarray Core Facility at the University of Colorado Anschutz Medical Campus for library preparation and sequencing. Library preparation and sequencing were performed with an Illumina TruSeq stranded mRNA sample preparation kit using standard Illumina HiSeq protocols and reagents.

### RNA sequencing data analysis.

Raw fastq files were assessed for quality via FastQC. 3′-End adapter sequences were trimmed from reads via Trimmomatic version 0.32. Raw fastq files were compared against human, mouse, and Escherichia coli genomes via fastqscreen version 0.5.2; no contaminations were found. Adapter-trimmed fastq files were then mapped back to hg19 reference genome via Tophat2 version 2.0.6 utilizing the hg19 gene annotations file (downloaded from UCSC genome browser database). After reads were mapped to hg19 reference genome, appropriate file conversions (from SAM format to BAM format) were made, including readName and position-based sorting via SAMtools version 0.1.16 for downstream analysis. Gene-level counts were obtained using HTseq version 0.6.1 (55), using the “stranded=reverse” and “intersection-nonempty” options, with annotation as described above. Only genes with values >0.5 counts per million in two samples were considered to be detected at levels sufficient for meaningful analysis. Principal-component analysis (PCA; R, Limma) of the 500 most-variable genes was used to visualize and assess variance associated with cell line, treatment, and sequencing batch, indicating a strong batch effect (see Table S2 in the supplemental material). Differential gene expression was determined using DEseq2 version 1.12.4 (56) in R (version 3.3.1), with sequencing batch information added to the generalized linear model to correct for batch effects, and a significance cutoff of a <0.1 adjusted *P* value. PCA plots, MA plots, and heat maps were made using the Python plotting library “matplotlib” (http://matplotlib.org/).

### Metascape analysis.

Pathway and process enrichment analysis for indicated gene sets was carried out using custom Metascape analysis (www.metascape.org) with the inclusion of reactome gene sets, canonical pathways (MSigDB), gene ontology biological processes, and Hallmark gene sets (MSigDB). Metascape determines statistically enriched terms using accumulative hypergeometric *P* values and clusters terms based on shared gene membership to reduce redundancy. Within each cluster the best *P* value is used to select a representative term for display and hierarchical clustering.

### GSEA.

Gene set enrichment analysis (GSEA) was carried out using the GSEA preranked module on the GenePattern server (57, 58), with log_2_ fold change values for all detected genes for the indicated comparisons as the ranking metric, and Hallmarks as the gene sets database to be tested for enrichment.

### Western blots.

Cells were harvested by scraping, rinsed with cold PBS, and suspended in radioimmunoprecipitation assay (RIPA) buffer complete with inhibitors. Lysates were sonicated in a Bioruptor and cleared with centrifugation. The protein concentration was determined using a Pierce BCA assay (Thermo Scientific). Samples were normalized to 2 mg/ml. Then, 20 to 40 μg of total protein was loaded per lane on an 8% denaturing polyacrylamide gel and transferred to polyvinylidene difluoride membrane. See Table S1C in the supplemental material for the primary antibodies used. Blots were imaged on an LAS4000 using Millipore chemiluminescent horseradish peroxidase substrate detection reagent.

### SJSA treatment and recovery.

Cells were plated and allowed to adhere and then treated with DMSO, 5-FU, or nutlin-3 as described previously (52). After treatment, the cells were rinsed with PBS, and the medium was replaced. Cortistatin A (100 nM) was administered 1 h prior to nutlin-3 treatment and replenished every 48 h. For counting, the cells were rinsed with PBS, trypsinized, and resuspended in fresh medium. The cell suspensions were mixed 1:1 with trypan blue and counted using a hemocytometer.

### Rescue experiments.

The wild-type CDK19 open reading frame (ORF) was subcloned into pcDNA3.1+ (GenScript), and wild-type CDK8 in pIRES2-EGFP was available in the Taatjes lab. SJSA cells (500,000) were plated and transfected with 5 μg of plasmid and 15 μg of polyethyleneimine (Sigma-Aldrich) and then counted at 24-h intervals. For the rescue of nutlin sensitivity, cells were treated with nutlin for 24 h at 24 h after transfection. The medium was changed, and the cells were counted at 24-h intervals.

### Site-directed mutagenesis.

Site-directed mutagenesis primers were designed using SnapGene (see Table S1D in the supplemental material). PCR was completed on the wild-type CDK19 ORF in pcDNA3.1+, and products were sent to Quintara Biosciences for sequence validation.

### ChIP.

Cells were fixed with 1.0% formaldehyde and harvested in RIPA buffer complete with inhibitors. Lysate (1.0 mg of total protein) was used with the indicated antibodies (see Table S1C in the supplemental material) and with 15 μl of protein G-Sepharose beads (GE Healthcare). For S2P and S5P antibodies, M-powder was bound to the protein G-Sepharose beads prior to immunoprecipitation. Chromatin immunoprecipitation (ChIP)-enriched DNA was analyzed by qPCR as described previously (59). ChIP primer sequences for the p21/CDKN1A locus are listed in Table S1E in the supplemental material.

### MVA treatment.

Control and CDK19 knockdown cells were cultured for 2 days in 1 mM concentrations each of mevalonic acid (MVA) and mevalonic acid phosphate (MVAP; Sigma-Aldrich [M4664 and 79849]) or left untreated. A total of 500,000 cells were plated in the presence or absence of MVA/MVAP and allowed to adhere before being treated with nutlin as described above. After nutlin treatment, the medium was changed, and the cells were allowed to recover in the presence or absence of MVA/MVAP. The medium was refreshed every 48 h, and the cells were counted as described above.

### Cholesterol quantitation.

Total cholesterol (cholesteryl esters and free cholesterol) concentration was measured using the Sigma-Aldrich cholesterol quantitation kit (MAK043). The cells were split the day prior to harvest; 10^6^ cells were harvested in 1.5-ml tubes, gently centrifuged, rinsed in PBS, and suspended in 200 μl of cholesterol extraction buffer (chloroform–isopropanol–NP-40, 7:11:0.1). The samples were mixed thoroughly by pipetting, vortexed, and homogenized using a micropestle. Samples were spun at 13,000 rpm for 10 min at room temperature, and the supernatant was transferred to a new 1.5-ml tube. After air drying at 50°C, the samples were vacuum dried for 30 min. The dried lipids were dissolved in cholesterol assay buffer and vortexed thoroughly until homogenous. The samples were tested at several dilutions, and the concentration was determined by measurement against standards.

### Accession number(s).

The accession number for the RNA-Seq data reported here is GEO GSE89807.

## Supplementary Material

Supplemental material
