# Supplementary material for: A Kinase-Independent Role for Cyclin-Dependent Kinase 19 in p53 Response
Source: Mol Cell Biol. 2017 Jun 15;37(13):e00626-16. doi: 10.1128/MCB.00626-16 (PMC5472832; doi:10.1128/MCB.00626-16)
Supplement: Supplemental material [file supp_37_13_e00626-16__index.html]

Supplemental material 

# A Kinase-Independent Role for Cyclin-Dependent Kinase 19 in p53 Response

## Supplemental material

- Supplemental file 1 -

  Table S1 (shRNA sequences)

  XLSX, 26K
- Supplemental file 2 -

  Table S2 (RNA-Seq analysis results)

  XLSX, 5.4M
- Supplemental file 3 -

  Table S3 (Gene expression changes)

  XLSX, 18M
- Supplemental file 4 -

  Table S4 (GSEA results)

  XLSX, 26K
- Supplemental file 5 -

  Table S5 (p53 pathway gene induction in shCTRL and shCDK19 cells)

  XLSX, 949K
